# Parametric investigation of a chilled water district cooling unit using mono and hybrid nanofluids

**DOI:** 10.1038/s41598-021-98754-7

**Published:** 2021-09-28

**Authors:** Eric C. Okonkwo, Tareq Al-Ansari

**Affiliations:** College of Science and Engineering, Hamad Bin Khalifa University, Qatar Foundation, Education City, Doha, Qatar

**Keywords:** Engineering, Energy infrastructure

## Abstract

This study presents a novel parametric investigation into the performance of a district cooling system using mono (Al_2_O_3_ and TiO_2_) and hybrid (Al_2_O_3_–TiO_2_) nanoparticles in the base fluids of water and ethylene–glycol water (EG-water) at a 20:80 ratio. The study analyses the effect of variables such as secondary fluid flow rate, evaporator and inlet temperatures, nanoparticle concentration, and air flowrate on the COP, total electrical energy consumption, and design of the district cooling unit. The analysis is performed with a thermal model developed and validated using operations data obtained from the McQuay chilled water HVAC unit operating in one of the facility plants at the Education City campus. The results of the study show that the use of nanofluids increased the overall heat transfer coefficient in the system by 6.6% when using Al_2_O_3_–TiO_2_/water nanofluids. The use of nanofluids in the evaporator also led to an average reduction of 23.3% in the total work input to the system and improved the COP of the system by 21.8%, 20.8% and 21.6% for Al_2_O_3_–TiO_2_/water, Al_2_O_3_/water, and TiO_2_/water nanofluids, respectively. Finally, an enhancement of 21.6% in COP was recorded for Al_2_O_3_–TiO_2_/EG-water nanofluids at a 5% nanoparticle volume concentration.

## Introduction

Effective thermal management in buildings (spaces) has led to advances in the design of air conditioning (AC) or refrigeration systems. The ventilation and thermal comfort of occupants in a space (residential or commercial) are of importance to engineers and scientists. Heating, ventilation, and air conditioning (HVAC) systems are used to achieve the required thermal and air quality levels for occupants in a building^1^. These district systems help in circulating indoor and outdoor air, by cooling or heating them before they are distributed into occupied spaces. The HVAC system can be utilised in any of the three processes, namely, the heating, cooling, and ventilation processes. The need to improve the operating conditions of the HVAC system has become paramount, as they account for large amounts of the total electricity consumption in buildings. The thermal management (heating or air conditioning) of residential spaces accounts for approximately 45%, 68%, and 35% of the electricity consumed in residential buildings in the United States, the European Union, and China, respectively^2^. While in commercial or service buildings, this number is at 39% globally^3^. Improving the efficiencies of the HVAC system would help decrease the energy consumption of the system and subsequently aid in the reduction of CO_2_ emissions from electricity generating plants. Amongst the three processes that can be performed by the HVAC system, this study focuses on the cooling process. Cooling is done by the HVAC with the aid of a chiller. Many of these chillers function using the processes associated with the traditional refrigeration system, where a refrigerant is circulated in a closed-loop comprising of a compressor, condenser, expansion valve, and evaporator^4^. At the evaporator, heat is absorbed by the refrigerant from a secondary fluid (usually water, air, or inhibitor glycol) which then circulates this cooled fluid to areas of the building where they are needed with the help of the air handling units (AHU) stationed in the buildings.

Nanofluids have been suggested as an alternative heat transfer fluid that would improve the rate of heat transfer and energy saving potentials of the HVAC system. Nanofluids are colloidal dispersion of nanoscale particles (10–100 nm) into a base fluid^5^. These nanoparticles can be metals, metal oxides, oxide ceramics, or carbon-based elements. The use of these nanoparticles helps improve the thermal and rheological properties of the base fluid and is beneficial for heat transfer purposes^6,7^. Nanofluids have tremendous potentials as next-generation heat transfer fluids in several heat transfer devices including solar collectors^8,9^, heat exchangers, radiators, heat pipes, electronic cooling, thermal storage, and HVAC systems. A detailed review of the application of nanofluids in these heat transfer devices has been carried out^10,11^.

Various studies have investigated the use of nanofluids in HVAC systems. Hatami et al.^12^, investigated the potential of three nanoparticles (CNT, TiO_2,_ and SiO_2_) for use in an HVAC system. Using the total dissolved solids (TDS) and the temperature of the heating area, they concluded that SiO_2_ was the best of the three nanoparticles tested due to their lower deposits and higher area temperature. In studies related to absorption refrigeration systems, Jiang et al.^13^, experimentally investigated the effect of TiO_2_ nanoparticles on the coefficient of performance (COP) of ammonia-water absorption refrigeration systems. By varying parameters like nanoparticle volume concentration (0.1–0.5%), evaporator temperature (− 18 to − 10 C) and generator temperature (22–33 C), the COP of the system was found to be enhanced by 27% at 0.5% volume concentration of nanoparticles. Pourfayaz et al.^14^, using silver water-based nanofluids as the absorbent liquid in an ammonia-water absorption chiller found that the presence of the nanoparticles increased the overall efficiency of their hybrid system by 81%. Researchers have also investigated the use of nanoparticles in active barocaloric cooling. These barocaloric refrigeration are a form of solid-state cooling systems that presents an eco-friendly alternative to vapour compression systems (VCS). Aprea et al.^15^, using ethylene glycol(EG) water (50–50%) with copper nanoparticles observed a moderate thermal performance enhancement of 4%, 7.3%, and 6.7% for the maximum temperature span, cooling power and COP of the system respectively. In another study^16^, Cu/ethylene glycol water (50–50%) was used as a secondary fluid in barocaloric refrigeration. The results showed that the addition of 10% Cu into EG-water gave an average of 30% enhancement in heat transfer within the system.

Nanofluids can also be used in vapour compression systems (VCS). Bhattad et al.^17^ in their review study highlighted that nanoparticles could be applied in vapour compression systems as nano-lubricants (nano-oil), nano-refrigerants, or/and as secondary fluids in the chilled water loop. As nano-lubricants, the effect of R134a/PAG oil-Al_2_O_3_ nanofluid on the COP, compression power, compressor discharge temperature, and refrigeration capacity has been investigated^18^. Varying the evaporator temperature (− 11 to 1 C) and condenser temperature (30 and 34 C) the nanoparticles showed a higher degree of subcooling at the condenser exit and improved COP by 6.5%. A marginal improvement of 1.5 C in refrigeration capacity and compressor discharge temperature was observed when using the nano-oil. Narendra and Rao^19^, using Al_2_O_3_ nanoparticles in compressor oil (Suniso-4GS oil) recorded an energy efficiency ratio (EER) increase of 0.13% while observing a COP enhancement of 3.8%. The performance of an air conditioning unit operating with SiO_2_/PAG nano-oil was investigated^20^. By varying the refrigerant charge between 95–125 g and compressor speed between 900–2100 RPM, they observed a maximum and average COP enhancement of 24% and 10.5%, respectively. The maximum enhancement was recorded at 0.05% volume concentration. Nano-lubricants would also help in reducing the pressure losses and power consumption in the system^17^.

The effect of nano-refrigerants on the performance of air-conditioning systems units has also been investigated^21^. Rahman et al.^22^ conducted an experimental study on a passive air-conditioning unit coupled with SWCNT/R-407c nano-refrigerants. While the cooling load of the system was decreased by 35% due to the use of trees, double glazed windows and roof reflective materials, the use of the nanoparticles decreased the power consumption of the air-conditioning unit by 202.5 W. Jeyakumar et al.^23^, investigated the effect of CuO, Al_2_O_3_ and ZnO nanoparticles on the COP of a refrigeration cycle. The nanoparticles were added to R134a refrigerant and the result showed an improved COP of 12.2% and 3.42% using CuO and Al_2_O_3_ nano-refrigerants, respectively as compared to conventional refrigerants with 0.1% polyester oil. They also observed a 1.39% and 0.6% reduction in power consumption when using CuO and Al_2_O_3_ nanoparticles, respectively. Bhattad et al.^17^ stated that nano-refrigerants had the potential to improve the boiling and condensation heat transfer coefficient which would lead to an improved and more compact vapour compression unit. They also highlighted that nanoparticles in the refrigerants would help improve the solubility between the refrigerants and lubricants leading to an enhanced compressor life span.

Nanofluids can also be used as secondary fluids in an HVAC system. Using a heat exchanger, the secondary fluid is used to transfer heat from the condenser or to the evaporator of the chillers. The effect of nanoparticles on the chilled water used in the evaporator has been investigated. Vasconcelos et al.^24^, investigated the performance of SWCNT in the secondary loop of a VCS. They varied the parameters of nanoparticle volume concentration (0–0.2%), nanofluid inlet temperature (30–40 C), and mass flowrate (40–80 kg/s) and found that the overall performance of the nanofluid was better than the base fluid (water). Ahmed et al.^25^, investigated the use of Al_2_O_3_ and TiO_2_ water-based nanofluids as chilled water in a vapour compression air conditioning unit. They analysed the effect of the hybridization ratio, nanoparticle type and concentration, fluid flow rate, and air velocity on the performance of the system. The results show that the Al_2_O_3_/water gave the highest COP at the least cooling time. A 5.3% and 4.1% improvement in refrigeration effect and reduction in compression ratio was recorded when using Al_2_O_3_/water as compared to TiO_2_/water nanofluids. Another study^26^, investigated the performance of a chill water AC unit with Al_2_O_3_/water as compared to pure water. The Al_2_O_3_/water was used in the cooling tank at 0.1, 0.2, 0.3, and 1% volume concentrations. Al_2_O_3_/water needed less time to achieve the desired temperature. A reduction in power consumption, an increase in cooling capacity, and enhanced COP were achieved by 5% and 17% when using 0.1% and 1% volume concentration of nanoparticles, respectively. Monirimanesh et al.^27^, evaluated the effect of TiO_2_/methanol nanofluids in a heat pipe heat exchanger on the temperature and relative humidity of an air conditioning system. The highest energy saving of 26.1–43.7% was observed with the nanofluid at a 3% volume concentration. Balaji et al.^28^, observed the effect of Al_2_O_3_/EG-water (30:70) on the compressor load of a domestic air conditioning system. They introduced a shell coil type heat exchanger to serve as an intercooler. The nanofluids were used in the shell side and the highest increment of 49.32% in COP was observed for the nanofluids at 0.75% concentration and a flow rate of 2 L/min, while a 12.2% reduction in power consumption was also recorded. The effectiveness and energy savings potential of methanol and silver/methanol nanofluids used in an HVAC unit were compared^29^. The results show that the nanofluid had an energy savings of about 8.1–31.5% and 18–100% when the supplied air stream in the HVAC unit is reheated. Alawi et al.^30^ after a review of nanofluid in refrigeration systems concluded that nano-refrigerants have the potential to decrease the energy consumption of the system, while the use of nano-lubricants has the potential to improve the rate of heat transfer with the system. Finally, they stated that the use of nanoparticles can help improve the COP and rate of cooling of the refrigeration cycle. Some other studies on the use of nanofluids in both the vapour compression and absorption systems are listed in Table 1.

**Table 1:** Studies related to the application of nanofluids in cooling systems.

Ref.	Nanofluid used	Method	Device	Used as	Key findings
^36^	MWCNT-CuO (30–70%)/SAE50	Experiment	Vapour compression system	Nano-lubricant	For high volume fractions (> 1%), the viscosity increased by 10%, which is less than the reported increase from other related studies on nanofluids
^37^	antifreeze-CoFe_2_O4/SiO_2_	Experiment	Vapour compression system	Nano-refrigerant	The nanofluids increased the performance of the system by 37.7%
^38^	graphene-acetone/ZnBr_2_	Experiment	Absorption refrigeration system	Absorbent	Nanoparticles improved boiling at lower temperatures
^39^	acetone/ZnBr_2_–ZnO	Experiment	Absorption refrigeration system	Absorbent	Acetone/ZnBr_2_ nanofluid improved the performance of this fluid in the vapour absorption refrigeration system
^40^	Al_2_O_3_–POE oil	Experiment	Vapour compression system	Nano-lubricant	Improved the COP of the system by 17.27%, and energy consumption was reduced by 32.48% at 0.1 vol% Al_2_O_3_ nanoparticles
^41^	CuO/water	Numerical	Solar absorption chiller	Absorbent	The application of the nanofluid enhanced the performance of the NH_3_–NaSCN and NH_3_–LiNO_3_ systems by 2.70% and 1.50%, respectively
^42^	TiO2-R600a nano-refrigerant	Experiment	Vapour compression system	Nano-refrigerant	The highest refrigeration effect was 290.83 kJ/kg for the R600a-TiO_2_ mixture and also had the highest COP value of 4.99

The use of nanofluids in HVAC has gained research attention over the past years. However, information on the performance of a new type of nanofluid that combines the properties of two or more nanoparticles known as hybrid nanofluids on HVAC systems is very scarce. Hybrid nanofluids have enhanced thermal and rheological properties due to the new chemical and physical bond formed within the fluid, these bonds result in better heat transfer characteristics for the resultant nanofluids^31–35^. Also, many studies on the HAVC system utilizes pure water-based nanofluids as a secondary fluid in the evaporator of the chiller. This, however, presents a technical restriction to their application, as water due to its freezing point is rarely used in chillers because of the possibilities of corrosion and icing in the flow channel. The commonly used fluids in these loops are inhibitor glycols (anti-freeze coolants) due to their much lower freezing points and anti-corrosion properties^1^. Studies using glycol-based nanofluids in this system for cooling are also rear in literature. On this basis, this study intends to fill these gaps and present a novel parametric investigation into the use of mono (Al_2_O_3_ and TiO_2_) and hybrid (Al_2_O_3_–TiO_2_) nanoparticles in the base fluids of water and ethylene glycol water (EG/water) at 20:80 ratio for use in an HVAC unit. The study considers the effect of variables such as the secondary fluid flow rate, evaporator and inlet temperatures, nanoparticle concentration, and air flowrate on the COP, total energy consumption, and design of the HVAC unit. The analysis is performed with a thermal model developed on the Engineering Equation Solver (EES). For the simulations of the nanofluids, their thermal properties and Nusselt number correlations are determined using equations from literature.

## System description and working fluids

### System description

**Figure 1:**
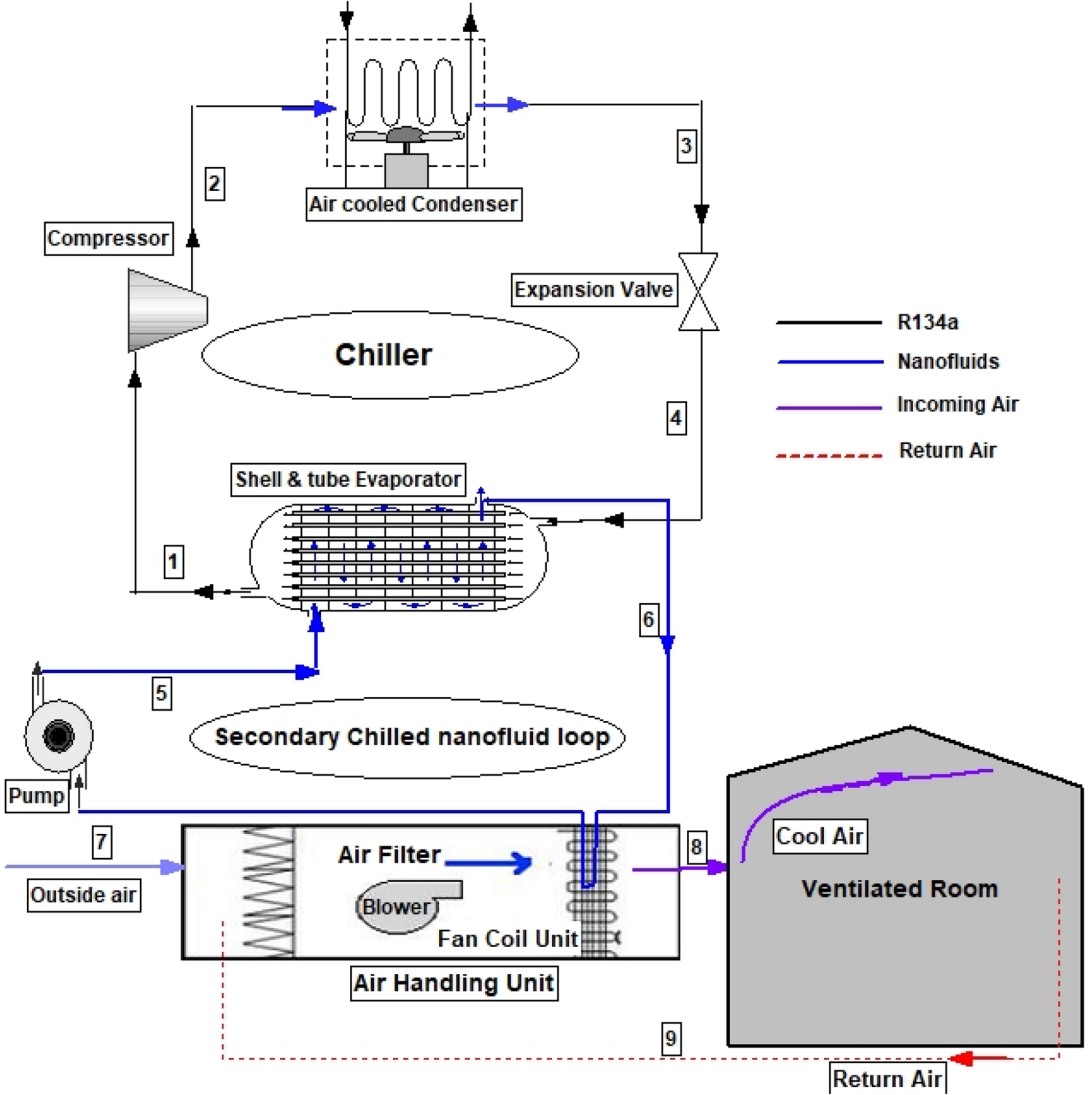
Schematic representation of the HVAC system.

Figure 1 shows a schematic representation of the air-cooled chilled water HVAC unit being studied. The unit consists of 3 loops namely, the chiller (first loop), the secondary fluid loop, and the air handling unit loop. The first loop is the typical vapour compression refrigeration system operating with R134a as a refrigerant. At state 1, saturated vapour undergoes isentropic compression at the compressor to give high temperature, high pressure (same as condenser pressure) superheated vapour. At state 2, the vapour enters the condenser at a temperature higher than that of the surrounding where with the help of a fan (air-cooled), heat is rejected to the surrounding at constant pressure. The refrigerant leaves the condenser at state 3 as saturated vapour before it is throttled down with an expansion valve to the evaporator pressure. The throttling process drops the temperature of the refrigerant to below 0 C levels, which are well below the temperature of the working fluid in the secondary loop. As the R134a refrigerant goes through the tube side of the shell and tube evaporator, it absorbs heat from the working fluid in the secondary loop (in this case water, EG-water, or nanofluids) flowing in the shell side of the evaporator, until it becomes saturates vapour at state 1 and the whole process repeats. The flow rate of the chilled working fluid (water, EG-water, nanofluids) is maintained by a pump and it transfers its energy to the air distribution system in the building which consists of a series of fans and coils connected by air ducts. The distribution system (air handling units (AHUs)) circulates filtered air from the ventilated space and outside air through the fan coil with the help of a blower into the ventilated space.

### Working fluids

This study evaluates the performance of eight different working fluids. Two different base fluids (water and EG-water at a ratio of 20:80 representing the percentage by volume of both fluids) are used and a comparison of their performance with Al_2_O_3_ and TiO_2_ nanoparticles in the base fluids are also investigated. The properties of the base fluids are obtained from the EES library, while that of the nanoparticles can be seen in Table 2.

**Table 2:** Thermal properties of nanoparticles^31,32^.

Parameters	Nanoparticles	
	TiO_2_	Al_2_O_3_
Specific heat capacity (J/kg·C)	690	773
Thermal Conductivity (W/m·C)	8.7	40
Density (kg/m^3^)	4010	3890

### Theoretical properties of the nanofluids

To calculate the properties of the nanofluids, the properties of the nanocomposite (Al_2_O_3_ + TiO_2_) must first be determined. The hybridization effects of the nanoparticles were studied at a ratio of 1:1 and each nanoparticle are represented by ${{\varphi }}_{1}$ for Al_2_O_3_ and ${{\varphi }}_{2}$ for TiO_2_. The total concentration of the hybrid nanoparticle is obtained using Eq. (1)^31^.

1 $${{\varphi }} = {{\varphi }}_{1} + {{\varphi }}_{2}$$

The equivalent equations for obtaining the thermophysical properties of density, specific heat capacity, and thermal conductivity of the resultant nanocomposite are given in Eqs. (2)–(4)^31^.

To obtain the density of the equivalent hybrid nanoparticle $\rho_{np}$ the Eq. (2) is used

2 $$\rho_{np} = \frac{{{{\varphi }}_{1} \cdot \rho_{np - 1} + {{\varphi }}_{2} \cdot \rho_{np - 2} }}{{{\varphi }}}$$

The specific heat capacity of the nanocomposite can be obtained using Eq. (3).

3 $$Cp_{np} = \frac{{{{\varphi }}_{1} \cdot \rho_{np - 1} \cdot Cp_{np - 1} + {{\varphi }}_{2} \cdot \rho_{np - 2} \cdot Cp_{np - 2} }}{{\rho_{np} \cdot {{\varphi }}}}$$

The thermal conductivity of the nanocomposite is given as:

4 $$k_{np} = \frac{{{{\varphi }}_{1} \cdot k_{np - 1} + {{\varphi }}_{2} \cdot k_{np - 2} }}{{{\varphi }}}$$

where the subscripts np-1 and np-2 represents the equivalent properties of Al_2_O_3_ and TiO_2_ nanoparticles, respectively.

The theoretical model for obtaining the density of the nanofluids which accounts for the variation in concentration at a constant temperature is given in Eq. (5)^43^.

5 $$\rho_{nf} = \rho_{bf} .\left( {1 - \varphi } \right) + \rho_{np} .\varphi$$

where the subscripts *nf*, *bf*, and *np* represent nanofluid, base fluid, and nanoparticles, respectively, while $\varphi$ represents the volume concentration of the nanoparticle.

The Eq. (6) below is used to theoretically obtain the specific heat capacity values of the nanofluid^43^.

6 $$C_{pnf} = \frac{{\rho_{bf} .\left( {1 - \varphi } \right)}}{{\rho_{nf} }}. C_{pbf} + \frac{{\rho_{np} .\varphi }}{{\rho_{nf} }}.C_{pnp}$$

The thermal conductivity of nanofluids is obtained using the Bruggeman model^44^. This equation is valid for spherical particles and considers the interaction between the particles.

7a $$k_{nf} = 0.25\cdot\left[ {\left( {3\varphi - 1} \right)k_{np} + \left( {2 - 3\varphi } \right)k_{bf} + \sqrt \Delta } \right]$$

7b $$\Delta = \left[ {\left( {3\varphi - 1} \right)k_{np} + \left( {2 - 3\varphi } \right)k_{bf} } \right]^{2} + 8k_{np} \cdot k_{bf}$$

The Brinkman model is used to calculate the dynamic viscosity of the nanofluids^45^.

8 $$\mu_{nf} = \mu_{bf} .\frac{1}{{\left( {1 - \varphi } \right)^{2.5} }}$$

The equation for viscosity is a general equation that is used to investigate all nanofluids. It should also be noted that Eqs. (5)–(8) account only for the variation in the base fluid properties and do not account for nanoparticle size or temperature. However, these limitations are the same for all examined nanofluids and are proper for the comparison of both mono and hybrid nanofluids.

## Theoretical modeling and simulation

The thermal modelling of the configuration in Fig. 1 is presented in this section. The system is modelled using the laws of conservation of energy, mass, and momentum. The dimensions and configurations of the key parameters are presented in Table 3. The system was modelled on the Engineering Equation Solver (EES) and the following assumptions are made.

- The system operates under steady-state conditions.
- Potential and kinetic energy changes in the fluid streams are negligible.
- Heat transfer in the evaporator heat exchanger is adiabatic.
- Both the flow of the refrigerant and secondary loop working fluids are fully developed.
- Uniform heat transfer occurs along the tubes of the evaporator heat exchanger.

**Table 3:** Dimensions and simulation parameters used in this study.

Parameter	Value	Unit
**Shell and tube evaporator design**		
Tube inner diameter (D_i_)	7	mm
Tube outer diameter (D_o_)	8	mm
Tube length (L)	2.7	m
Number of tubes (N_t_)	24	–
Tube layout pitch (P_T_)	8	mm
Baffle spacing (B)	50	mm
Thermal conductivity of steel tube (k_t_)	50	W/m-C
Number of passes	Single	–
Baffle cut (c_b_)	25%	
Inner diameter of the shell	0.4572	m
**Fan coil unit design**		
Heat transfer area on the air side of the coil	33	m^2^
Heat transfer area on the water side of the coil	1.67	m^2^
Heat transfer coefficient on the air side of the coil	284	W/m^2^C
Fan blower speed	3500	rpm
**Simulation parameters of this study**		
Pressure in secondary loop/ blower air inlet	101	kPa
Ambient/blower air inlet temperature	25	C
The outlet temperature of secondary fluid (T_6_)	6	C
Condenser exit temperature (T_3_)	27	C
Electrical efficiency of fan and blower ($\eta_{elect}$)	60%	

The general mass balance equation for each element is described as follows

9 $$\dot{m}_{in} = \dot{m}_{out}$$

The general form of the energy balance equation used for any component can be written as follows.

10 $$\Delta E = \dot{E}_{in} - \dot{E}_{out}$$

11 $$\Delta \dot{E} = \dot{Q} - \dot{W} + \sum \dot{m}_{in} h_{in} - \sum \dot{m}_{o} h_{o}$$

where $\Delta E$ is the change in energy of a system, and this value is zero for any system operating under steady-state conditions. $\dot{Q}$ and $\dot{W}$ represents the heat transfer and work rates within a system. The mass flow rate and specific enthalpy at each state point is represented by $\dot{m}$ and $\dot{h}$ respectively.

### Evaporator heat exchanger equations

Since the focus of the study is to investigate, the effect of using various nanofluids as a coolant in the shell and tube evaporator, detailed modelling of this component is needed to account for all possible thermal interaction within the unit. The refrigerant R134a enters and exits from the tube side of the heat exchanger, while the secondary loop working fluid (water, EG-water (20:80), or nanofluids) flows through the shell side of the heat exchanger.

An expression for the rate of heat transfer in the evaporator is given by

12 $$\dot{Q} = UA\Delta T_{LMTD}$$

13 $$\Delta T_{LMTD} = F \frac{{\Delta T_{1} - \Delta T_{2} }}{{\ln \left( {\frac{{\Delta T_{1} }}{{\Delta T_{2} }}} \right)}}$$

where *F* is the correction factor, which depends on the geometry of the heat exchanger and the inlet and outlet temperatures of the hot and cold fluid streams. Due to the condensation of the R134a refrigerant in the tube side of the heat exchanger, the value of *F* is 1. In this study, the hot fluid (*T*_*h*_) is the nanofluids and the cold fluid (*T*_*c*_) is the R134a refrigerant. Also, $\Delta T_{1} = T_{hi} - T_{co}$ and $\Delta T_{2} = T_{ho} - T_{ci}$.

The overall heat transfer coefficient (*U*) and the total heat transfer area (*A*_*o*_) are determined by the following equations^46^.

14 $$\frac{1}{{U_{o} }} = \frac{{D_{o} }}{{D_{i} h_{i} }} + \frac{{D_{o} \ln \frac{{D_{o} }}{{D_{i} }}}}{{2k_{t} }} + \frac{1}{{h_{o} }}$$

15 $$A_{o} = \pi D_{o} N_{t} L$$

The heat transfer relationship between the two working fluids in the evaporator heat exchanger can be expressed as

16 $$\dot{Q} = \dot{m}_{c} \left( {h_{1} - h_{4} } \right) = \dot{m}_{h} c_{ph} \left( {T_{5} - T_{6} } \right)$$

The coefficient of heat transfer $h_{o}$ and $h_{i}$ on both sides of the heat exchanger are obtained based on the geometry of the tube, fluid flowrate, and properties of the fluid^46,47^.

17 $$h_{o} = \frac{{Nu_{h} D_{s} }}{{k_{h} }}$$

18 $$Re_{h} = \frac{{\dot{m} D_{o} }}{{A_{s} \mu_{h} }}$$

19 $$Pr_{h} = \frac{{c_{ph} \mu_{h} }}{{k_{h} }}$$

20 $$A_{s} = D_{s} \times B \times \left( {1 - D_{o} /P_{T} } \right)$$

21 $$D_{s} = \left( {1.1 \times \sqrt {N_{t} } - 1} \right)P_{T} + 3D_{o}$$

The pressure drop in the shell side ($\Delta P_{s}$) is obtained using the equations from Bell-Delaware^48^. This equation accounts for the pressure losses in the central cross flow section, the window section, and the entrance and exit sections of the shell. These equations are presented in (22)–(24).

22 $$\Delta P_{s} = \left[ {\left( {\frac{L}{B} - 1} \right)\Delta P_{bi} R_{b} + \left( {\frac{L}{B} - \frac{1}{2}} \right)\Delta P_{wi} } \right]R_{l} + \Delta P_{bi} \left( {\frac{{1 - 1.2c_{b} }}{{1 - 2c_{b} }}} \right)R_{b} R_{s}$$

where the pressure drops in central ($\Delta P_{bi}$) and window ($\Delta P_{wi}$) sections are given by Eqs. (23)–(25) and *R*_*b*_, *R*_*s*_, *R*_*l*_ are given as 0.6, 1.0 and 0.4 respectively^48^.

23 $$\Delta P_{bi} = \frac{2f}{{\rho_{h} }}\left( {\frac{{\dot{m}_{h} }}{{A_{s} }}} \right)^{2} \left( {\frac{{\mu_{h,t} }}{{\mu_{h} }}} \right)^{0.14}$$

24 $$\Delta P_{wi} = \frac{{\dot{m}_{h}^{2} \left( {2 + 0.554c_{b} D_{s} /P_{T} } \right)}}{{2 \rho_{h} A_{s} A_{w} }}$$

where the area of the flow-through window $A_{w} = 1.04 A_{s}$ and the friction factor is given as^49^

25 $$f = \exp \left( {0.576 - 0.19\ln Re_{h} } \right) + C_{f}$$

where $C_{f}$ is 0.2873 for $Re_{h}$ ≤ 10^4^ and $C_{f}$ = 0.129for $Re_{h}$ ≥ 10^4^^49^

The Nusselt number (Nu) correlation for the Al_2_O_3_ or TiO_2_ nanofluids are calculated using the Pak and Cho correlation^50^

26 $$Nu = 0.021Re^{0.8} Pr^{0.5} ,\quad {\text{For 6}}.{54} \le Pr \le { 12}.{3};{ 1}0^{{4}} \le Re \le { 1}0^{{5}}$$

The Nusselt number in the shell side of the heat exchanger with a 25% baffle cut is calculated using Kern’s correlation^51^.

27 $$Nu{ } = { }0.472{ }.{ }Re^{ - 0.477} { }.{ }Re.{\text{ Pr}}^{0.33}$$

The correlation is valid for 10 < *Re* < 10^6^ and provided the best agreement with the experimental results compared to other theoretical correlations.

The mass flow rate defines the rate at which the fluids passes through the shell or tube and it is expressed as:

28 $$\dot{m} = A_{s} \cdot \rho \cdot v$$

where $v$ is the velocity of the fluid flowing inside the tube.

Another parameter used in this study to expresses the flow rate is the volume flow rate (L/min) and it is expressed as:

29 $$V_{f} = 60,000 \cdot u \cdot A_{s}$$

### Cooling coil

The cooling energy from the shell and tube evaporator, ventilated space cooling capacity from the fan coil unit (FCU) is obtained using Eqs. (30)–(35).

30 $$\dot{Q}_{evp } = \dot{m}_{c} \left( {h_{1} - h_{4} } \right)$$

31 $$\dot{Q}_{air,cool} = \dot{m}_{air} \left( {h_{8} - \left( {h_{7} + h_{9} } \right)} \right) + \left( {\dot{m}_{h} h_{condensate} } \right)$$

where $h_{condensate}$ is obtained at the enthalpy of vaporization of the secondary working fluid at *T*_*8*_, which equals zero in this study.

The nanofluid local heat transfer coefficient is calculated using the following equation:

32 $${\text{h}}_{{{\text{i}},{\text{ coil}}}} = { }\left( {{\text{Nu}}_{{{\text{i}},{\text{coil}}}} { } \times {\text{ k}}} \right)/{\text{D}}_{{\text{t}}}$$

The Nusselt number for turbulent flow valid for Reynold numbers between 2300 to 5 × 10^6^ and Prandtl number between 0.5 to 2000 is obtained from the following equation^52^:

33 $${\text{Nu}}_{{{\text{i}},{\text{ coil}}}} = \frac{{\left( {\frac{{f_{t} }}{8}} \right) \times \left( {Re_{t} - 1000} \right) \times Pr }}{{1 + 12.7 \times \left( {\frac{{f_{t} }}{8}} \right)^{0.5} \times \left( {Pr^{\frac{2}{3}} - 1} \right)}}$$

The friction factor is presented in Eq. (30)^52^.

34 $$f_{t} = \frac{1}{{0.79 \times ln\left( {Re_{t} - 1.64} \right)^{2} }}$$

The pressure drop in the cooling coil tube is calculated using Eq. (35)^52^.

35 $$\vartriangle p_{t} = \left( {4.f_{t} .\left( {\frac{{L_{t} }}{{D_{t} }}} \right) + 4} \right).\left( {\rho \times \frac{{v_{t}^{2} }}{2}} \right)$$

### Work input

The work done by the system is the sum of the work done by the pump ($\dot{W}_{p}$) and the compressor ($\dot{W}_{c}$), these work equations are presented in Eqs. (36)–(38).

36 $$\dot{W}_{c} = \dot{m}_{c} \left( {h_{2} - h_{1} } \right)$$

The pump work in the chilled water loop is expressed as;

37 $$\dot{W}_{p} = \left( {\frac{{\dot{m}_{h} }}{{\rho_{h} }}\Delta P_{s} } \right)/\eta_{elect}$$

The total work in the system is expressed as;

38 $$\dot{W}_{total} = \dot{W}_{p} + \dot{W}_{c} + \dot{W}_{blower}$$

The ensemble coefficient of performance of the HVAC system is evaluated using.

39 $$COP = \frac{{\dot{Q}_{air, cool} }}{{\dot{W}_{total} }}$$

## Validation

**Figure 2:**
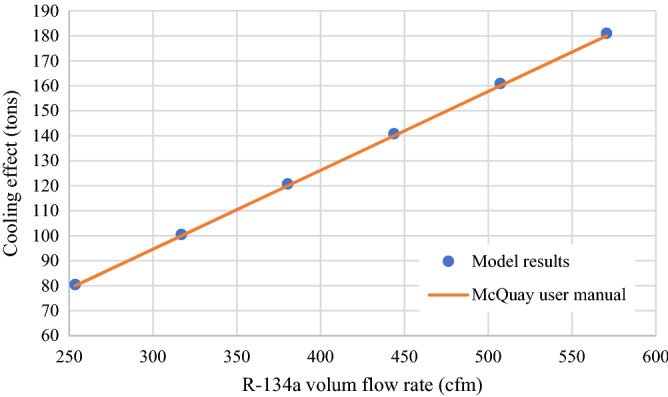
Accuracy of the model results compared to manufacturer results.

The HVAC system was designed based on the design specifications provided by the McQuay centrifugal compressor water chillers. The manual of the McQuay system provided specific design parameters for the chiller with an evaporator pressure of 124.1 psi (855.7 kPa) and condenser pressure of 35 psi (241.3 kPa)^53^. For the given pressures, the temperatures at both evaporator and condenser can be calculated. The design parameter of the chiller was used as input values for the present model. The manufacturer specified that for the stated pressures, the flow rate of the refrigerant controls the cooling output of the chiller. The results of varying refrigerant flow rates on the cooling outputs for both the plant and the model are presented in Fig. 2. The model results show a good agreement with the operational results with a percentage error of less than 0.6%.

## Results and discussion

This section evaluates the effects of the base fluids, along with the mono and hybrid nanofluids on the performance of the HVAC unit. The effect of the parameters of nanoparticle volumetric concentration, volume flow rate, and operating temperatures of the evaporator and secondary loop on the system’s performance is analytically investigated. These parameters are outlined in Table 4, along with the operating range considered in this study. The overall heat transfer coefficient, cooling energy, total work input, and COP of the HVAC are amongst the key performance outputs studied. Finally, an optimization of the key parameters listed in Table 4 is performed to obtain the optimum conditions of these parameters to achieve the maximum COP for each working fluid. In this study, the nanoparticle volume concentration is limited to 5%, as there are limited experimental studies on the behaviour of nanofluids beyond the volume concentration of 5% and it has been ascertained that higher concentrations of nanoparticles affect the stability of the nanofluids^14,42^. Also, the accuracy of Eqs. (5)–(8) are higher for lower values of nanoparticle fraction^14^.

**Table 4:** Parameters investigated in the HVAC model.

Parameter	Unit	Ranges		Classification
		Lower bounds	Upper bounds	
Nanoparticle volume concentration	%	0	5	User-specified
Secondary fluid mass flow rate	kg/s	1	26	User-specified
Evaporator temperature	C	− 40	0	User-specified
Secondary fluid inlet temperature (T_5_)	C	10	30	Operational
Air flow rate	m^3^/s	0.27	6.2	Operational

### Effect of studied parameters on the overall heat transfer coefficient in the HVAC evaporator

**Figure 3:**
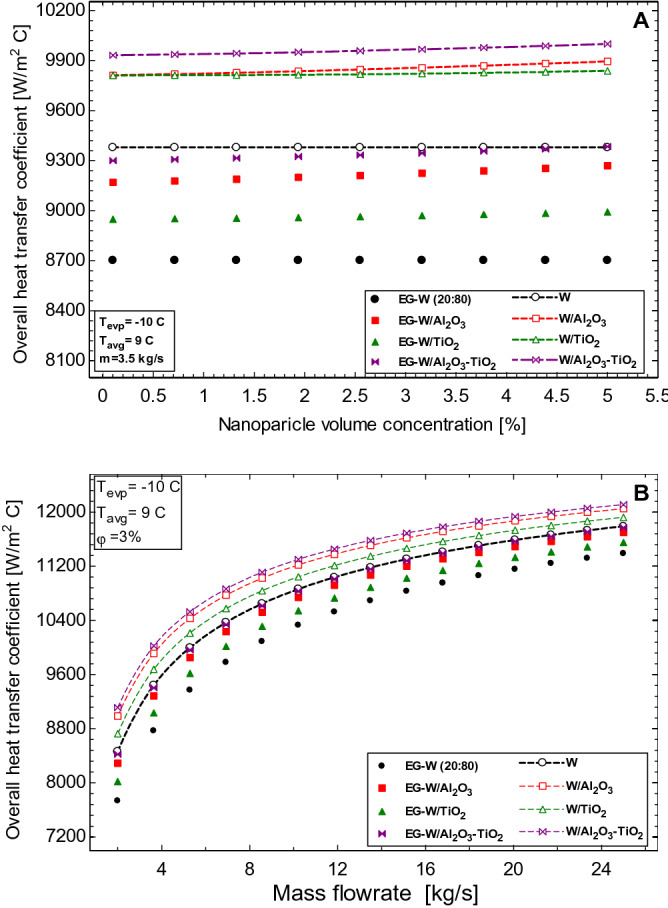
Effect of (**A**) nanoparticle volume concentration and (**B**) mass flowrate on the overall heat transfer coefficient of the fluids inside the evaporator.

The overall heat transfer coefficient in the evaporator is an important factor that determines the cooling effect obtained from the HVAC unit. This factor accounts for the individual heat transfer coefficient in the tube and shell sides of the evaporator. As seen in Eqs. (14)–(21), this parameter is a function of the properties of the working fluids on both sides of the evaporator heat exchanger, the mass flow rate of the working fluid, and the dimensions of the heat transfer surface in the evaporator. As the working fluid (R134a) flowing in the tube side of the evaporator is the same for all secondary fluids considered, the value of *h*_*i*_ in the tube side will be the same for all considered working fluids in the shell side. The effect of using nanoparticles in water and EG-water solution is seen in Fig. 3. The presence of nanoparticles enhances the properties of the base fluids hence the resultant nanofluids have a much higher overall heat transfer coefficient than the base fluids (Fig. 3A). The water-based nanofluids perform better than the EG-water nanofluids because they possess much higher thermal conductivity and lower viscosity values.

The highest enhancement of 7.8% in the overall heat transfer coefficient is seen with the use of Al_2_O_3_–TiO_2_/EG-water based nanofluids, while the Al_2_O_3_/EG-water and TiO_2_/EG-water nanofluids had 6.5% and 3.3% respectively. The enhancement recorded for the water-based nanofluids were 6.6%, 5.5%, and 2.7% for the Al_2_O_3_–TiO_2_/water, Al_2_O_3_/water, and TiO_2_/water nanofluids, respectively. All values are obtained at a flow rate of 200 L/min and nanoparticle concentrations of 5%.

**Figure 4:**
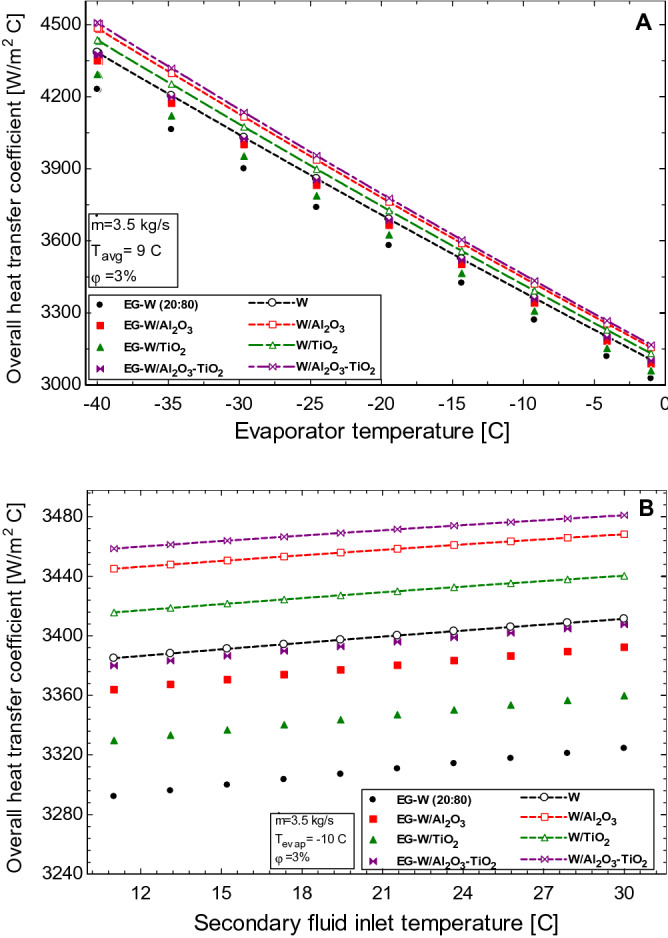
Effect of (**A**) evaporator temperature and (**B**) secondary fluid inlet temperature on the overall heat transfer coefficient in the evaporator.

The mass flow rate of the working fluid tends to favour the overall heat transfer coefficient in the evaporator as seen in Fig. 3B. The increase in the volume flow rate increases the Reynolds number of the flow stream and this results in a higher rate of heat transfer to the outer walls of the evaporator. However, the logarithmic nature of Fig. 3B shows that there exists an optimum flow rate after which further increase in flow rate will result in no significant enhancement in the overall heat transfer coefficient. Figure 3B also shows that the nanofluids performed relatively better than the corresponding base fluids although the effects of the nanoparticles are seen to decrease as the mass flow rate increases. This is due to the increased effect of turbulent flow as a result of the fluid flow rate exceeding the benefits of the fluid property from the use of the nanofluids. The Al_2_O_3_–TiO_2_ hybrid nanofluids are seen to show the best performance ahead of Al_2_O_3_ and TiO_2_ nanofluids, respectively. Figure 4 shows the effect of the evaporator temperature (Fig. 4A) and the secondary fluid inlet temperature (Fig. 4B) on the overall heat transfer coefficient in the evaporator. The evaporator temperature represents the temperature of the refrigerant (in this case R134a) in the tube side of the evaporator heat exchanger. This temperature is fixed in most chillers but varies depending on the working fluid (refrigerant) used in the chiller as different refrigerants have different evaporative temperatures and can range anywhere between − 40 to 0 C*.* From Fig. 4A, it is observed that the value of the overall heat transfer coefficient decreases with an increase in the evaporator temperature. This is because the logarithmic temperature difference is greater at lower evaporator temperatures and this decreases as the temperature of the evaporator increases. Although the overall heat transfer coefficient in the evaporator decreases, the enhanced performance of the nanofluids is obvious from the figure. However, an inverse effect is seen when the inlet temperature of the secondary working fluid is increased (Fig. 4B). The overall heat transfer coefficient is generally improved for all working fluids because the properties of the working fluids are temperature-dependent and these fluids become less viscous at higher temperatures, resulting in higher values of Reynolds number (Eq. 18) and hence better heat transfer results.

### Effect of studied parameters on the cooling output of the HVAC unit

**Figure 5:**
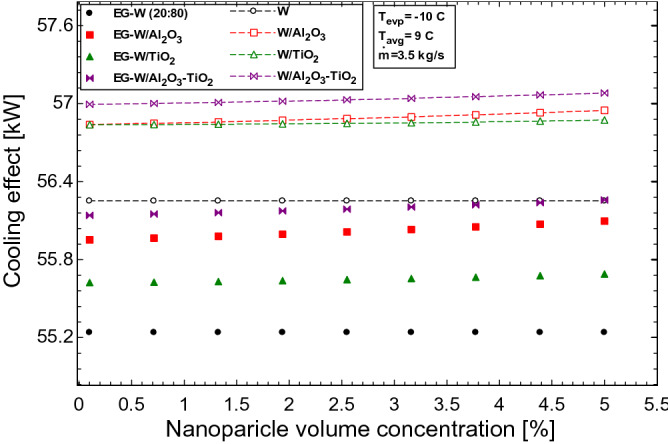
Effect of nanoparticle volume concentration on the cooling effect of the HVAC.

The effects of the studied parameters on the characteristic performance of the HVAC unit is analysed as a function of the cooling effect. The cooling effect reflects the quantity of energy used in cooling the ventilated space. In this section, this energy is represented in kilowatt (kW) or tons of refrigeration (R Ton), where 1 R Ton = 3.516 kW. Figure 5 shows the impact of nanoparticle loading on the cooling effect obtained from the HVAC unit. The cooling effect obtained from the nanofluids is seen to marginally increase, with the use of Al_2_O_3_–TiO_2_ hybrid nanofluids showing the best performance for both the water and EG-water base nanofluids, respectively. The cooling output is increased due to the Brownian motion of particles in the nanofluids as the concentration of nanoparticles is increased. This random motion of particles aid in the thermal transport mechanism within each nanofluid. The more thermally enhanced hybrid nanofluids perform better due to the enhanced thermal conductivity properties of the nanoparticles as compared to Al_2_O_3_ and TiO_2_ nanofluids, respectively. Also, the higher thermal properties of water as compared to EG-water mean that the resulting water base nanofluids have better thermal properties than that of the EG-water nanofluids. The enhancement in cooling effect as a result of using nanofluids accounts for 1.5%, 1.2%, and 0.6% for Al_2_O_3_–TiO_2_, Al_2_O_3_, and TiO_2_ water-based nanofluids respectively, while 1.8%, 1.5% and 0.8% were witnessed for the Al_2_O_3_–TiO_2_, Al_2_O_3_, and TiO_2_ EG-water based nanofluids respectively at 5% nanoparticle concentration.

**Figure 6:**
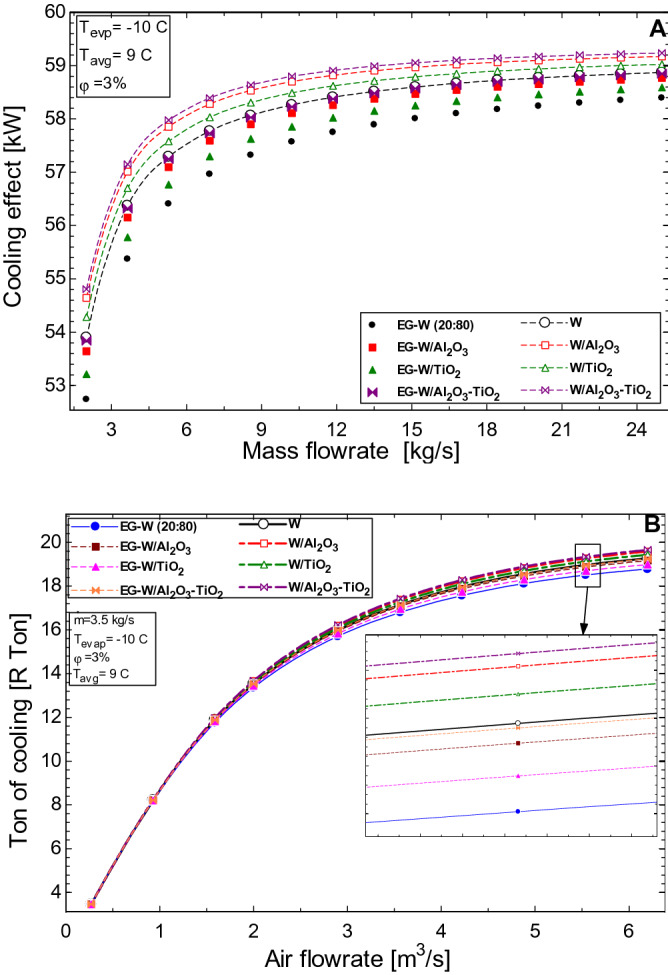
Effect of (**A**) mass flow rate of working fluids and (**B**) air flow rate on the cooling effect of the HVAC.

The effect of the mass flow rate on both the working fluids in the secondary loop and the air flow rate across the fan coil unit is seen in Fig. 6. Similar to the trend observed in Fig. 3B, the effect of increasing the flow rate of the secondary fluid results in an increase in the cooling effect from the HVAC (Fig. 6A). The less viscous water and its nanofluids are seen to perform better than the EG-water solution. The enhanced properties of the nanofluids result in a better performance than their respective base fluids at the same flow rate. The air flow rate in the fan coil unit (Fig. 6B) shows, a similar trend to Fig. 6A. In both cases (FCU and evaporator), the convection coefficient is seen to increase with an increase in the flow rate across both heat exchangers. This increase would result in a higher cooling return from the HVAC unit. As shown in Fig. 6B, the cooling capacity is increased from 3.4 R Ton to over 19 R Ton as the airflow rate is adjusted from 0.2 to 6.2 m^3^/s. An average airflow rate of 0.16–0.18 m^3^/s is needed for 1 R Ton of cooling.

### Effect of studied parameters on the work input to the HVAC unit

**Figure 7:**
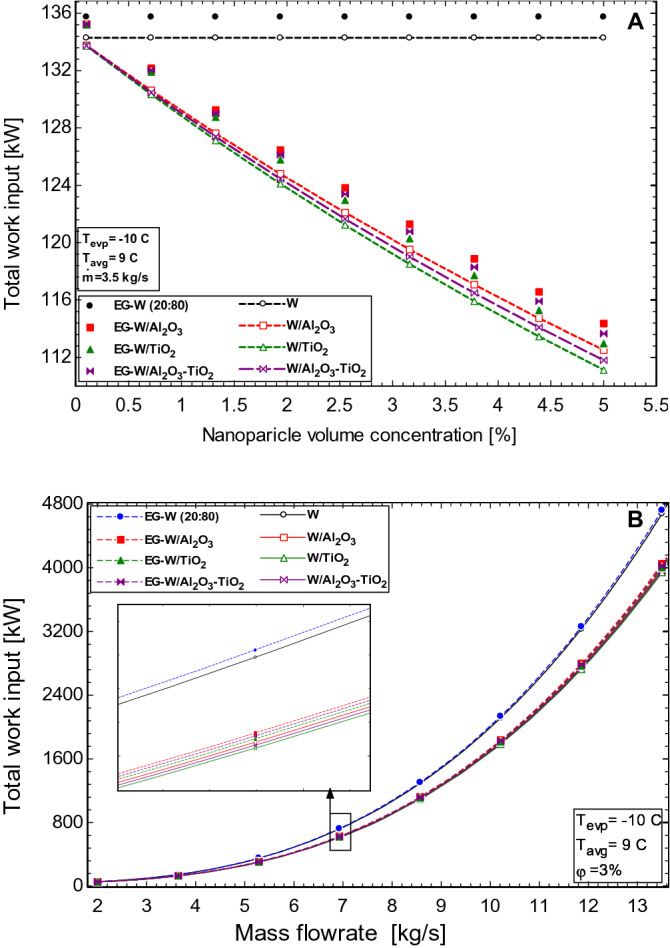
Effect of (**A**) nanoparticle volume concentration and (**B**) volume flowrate of working fluids on the work input of the HVAC.

Figure 7A shows that the work done by the HVAC is decreased with nanoparticle loading. The major contributing factor to the work in the HVAC comes from the pump used to maintain the flowrate in the secondary loop. Figure 7A shows that the EG-water based nanofluids require more pump power than the water-based nanofluids, this is because of the much higher viscosity of EG-water as compared to pure water. At a constant mass flow rate, the use of nanofluids benefits the system, as less work is required by the pumps. An average of 23.29% reduction in the total work of the HVAC unit is achieved when using the nanoparticles at a volume concentration of 5%.

Figure 7B shows the effect of the mass flow rate on the total work input in the HVAC. The pressure losses in the secondary loop are strongly correlated to the required pump work into the system. As the pressure losses increase due to an increase in flow rate, the amount of work required by the pump also increases. This influences the overall performance of the HVAC as seen with Eqs. (34) and (35). The water-based nanofluids as explained earlier are significantly less viscous than the EG-water nanofluids and hence witness much fewer pressure losses and pump work.

**Figure 8:**
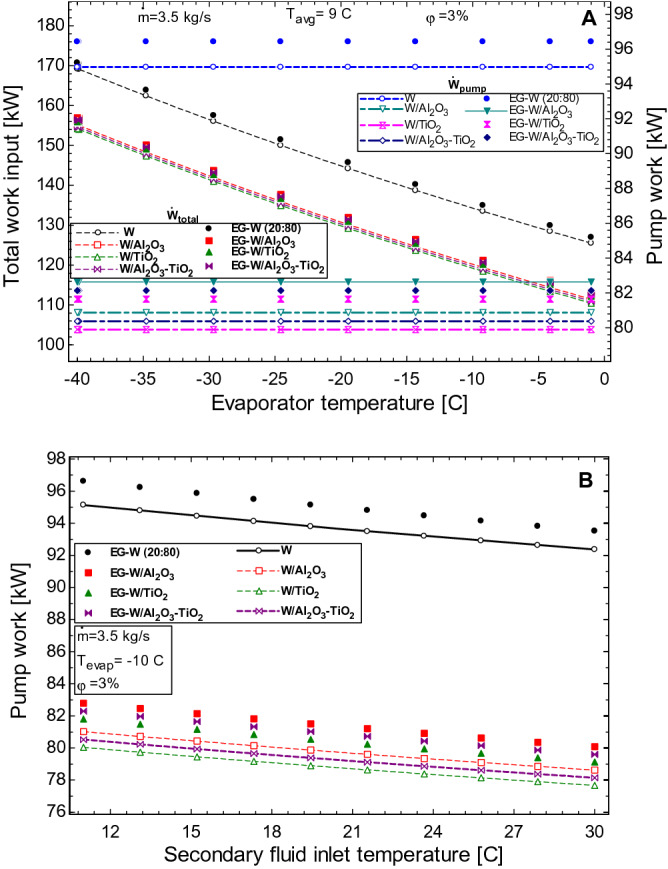
Effect of (**A**) evaporator temperature and (**B**) secondary fluid inlet temperature on the pump work of the HVAC.

The effect of the evaporator temperature and inlet temperature of the working fluids on the pump work of the HVAC is seen in Fig. 8. The total work required by the HVAC is decreased with an increase in the evaporator temperature (Fig. 8A). It should be noted that this reduction in the work input into the system is largely due to a reduction in the compression work as the temperature of the evaporator increases. This reduction is due to the decrease in the temperature and pressure difference between states 1 and state 2. As can be seen from Fig. 8A, the pump work remains the same for all working fluids even as the evaporator temperature changes. The effect of the inlet temperature change on the pump work of the secondary loop is seen in Fig. 8B. The pump power required to maintain flow is seen to decrease as the inlet temperature increases owing to the reduction in the viscosity of the various working fluid with rising temperatures. This shows that the effect of fluid friction is minimized, and the pressure losses are decreased leading to a decrease in the pump requirements of the system.

### Effect of studied parameters on the coefficient of performance of the HVAC unit

**Figure 9:**
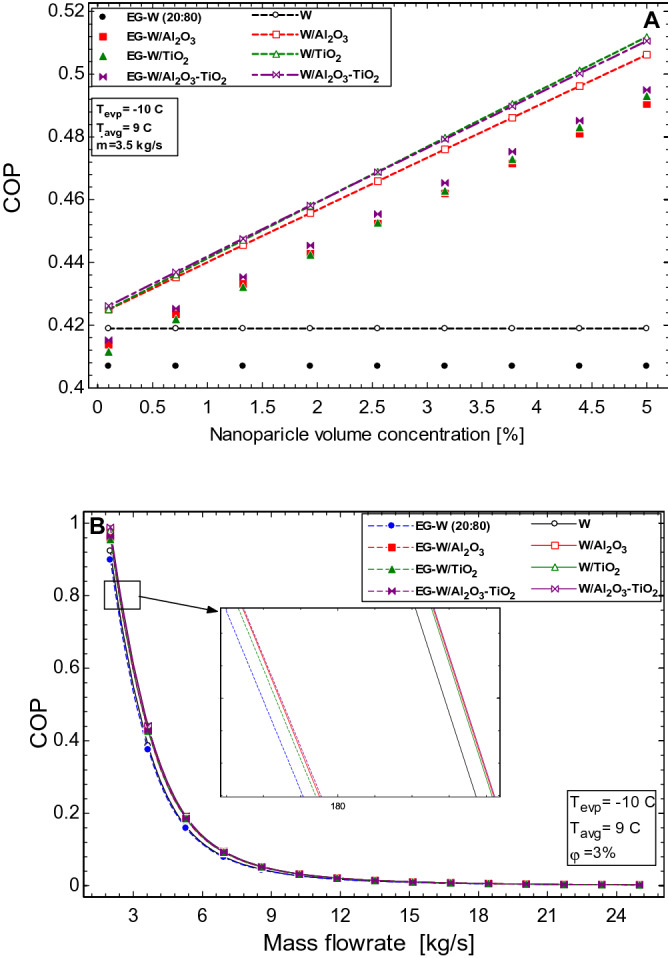
Effect of (**A**) nanoparticle volume concentration and (**B**) mass flow rate of working fluids on the COP of the HVAC.

**Figure 10:**
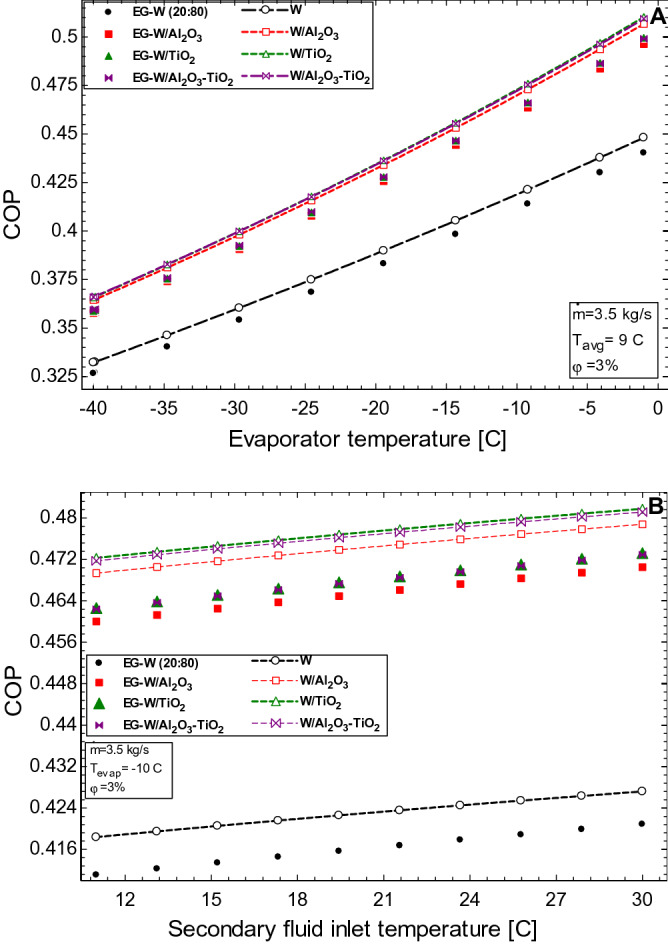
Effect of (**A**) evaporator temperature and (**B**) secondary fluid inlet temperature on the COP of the HVAC.

The effect of the nanoparticle volume concentration on the COP of the system is seen in Fig. 9A. It is observed that an increase in nanoparticle loading increases the COP of the HVAC. The most beneficial concentration of nanoparticle for the flow rate considered is 5%, and the COP is seen to improve for both sets of the nanofluids studied. The enhancement in the water-based nanofluids are higher than those witnessed in the EG-water nanofluids, the Al_2_O_3_–TiO_2_/water nanofluid showed the best performance with a 21.9% enhancement while Al_2_O_3_/water and TiO_2_/water nanofluids had 20.8% and 21.6% enhancement, respectively. For the EG-water based nanofluids, the Al_2_O_3_–TiO_2_/EG-water nanofluid showed the best performance with a 21.6% enhancement while Al_2_O_3_/EG-water and TiO_2_/EG-water nanofluids witnessed a 20.5% and 21.2% enhancement, respectively. The COP performance for changing flowrate is seen in Fig. 9B. The decrease in COP is attributed to an increase in the power requirement of the HVAC system. This parameter affects all the working fluids studied, however, the nanofluids are still seen to perform better than the base fluids. Figure 10 shows the impact of the evaporator and inlet temperatures on the COP of the HVAC system. From Fig. 10A, the COP is increased as the evaporator temperature increases. A rule of thumb is that the COP improves by 2 to 4% for each C the evaporating temperature is raised. This is because an increase in the evaporator temperature increases the evaporator pressure and therefore less work is required by the compressor. Also, in Fig. 10B, the pump work decreases with an increase in the inlet temperature of the secondary fluids, and this favour an increase in the COP of the system.

The use of nanofluids has the potential to impact the design of parts of the HVAC system. This is supported by Eq. (12), which proves that at a constant rate of heat transfer, the use of nanofluids can lead to a reduction in the required heat transfer surface of the heat exchanger. This outcome can result in improvements in the design of the size and volume of the evaporator used in the chiller.

## Conclusion

This study presents a novel parametric investigation into the use of mono and hybrid nanoparticles in the chilled water loop of a district cooling unit. The study analysed the effect of several variables on the COP, total energy consumption and design of the HVAC district cooling unit. The analysis is performed using a thermal model developed on the Engineering Equation Solver (EES) and validated with operations data obtained from a McQuay chilled water unit operating in one of the facility plants at the Education City campus of Qatar Foundation. From the findings, the following conclusions are made:

- The use of nanofluids was found to increase the rate of heat transfer in the HVAC system. The rate of cooling was significantly increased by adding the nanoparticles into the base fluids.
- Within the range of nanoparticle concentration used in this study, it was discovered that increasing the volume concentration of nanoparticles in all the base fluid studied improves the performance of the district cooling system.
- At a nanoparticle volume concentration of 5%, the use of nanofluids led to a 23.3% average reduction in the pump work of the district cooling system. The highest reduction in additional pump work recorded was 24.4% for TiO_2_/water nanofluid while the least observed was 22.18%, which was, recorded when Al_2_O_3_ /EG-water nanofluid is used.
- The overall heat transfer coefficient in the district cooling unit was increased by using the nanofluids. At a 5% volume concentration, a 6.6% enhancement in this parameter was recorded for Al_2_O_3_–TiO_2_/water nanofluid while 5.5% and 2.7% were obtained when using Al_2_O_3_/water and TiO_2_/water nanofluids, respectively. An enhancement of 7.8%, 6.5% and 3.3% was recorded when using Al_2_O_3_–TiO_2_, Al_2_O_3_, and TiO_2_ EG-water nanofluids, respectively.
- The use of nanofluids in the evaporator improved the coefficient of performance of the district cooling system. Al_2_O_3_–TiO_2_/water nanofluid had the best performance with a 21.8% enhancement in COP while an increase of 20.8% and 21.6% were achieved with Al_2_O_3_/water and TiO_2_/water nanofluids, respectively.
- The use of the hybrid nanofluids also recorded the highest enhancement in COP for the EG-water based nanofluids, as an enhancement of 21.6%, 20.5%, and 21.1% were recorded for Al_2_O_3_–TiO_2_/EG-water, Al_2_O_3_/EG-water, and TiO_2_/EG-water nanofluids, respectively.
- The enhancement in cooling effect as a result of using nanofluids accounts for 1.47%, 1.24% and 0.63% for Al_2_O_3_–TiO_2_, Al_2_O_3_, and TiO_2_ water-based nanofluids respectively, while 1.84%, 1.54%, and 0.81% were witnessed for the Al_2_O_3_–TiO_2_, Al_2_O_3_, and TiO_2_/EG-water nanofluids respectively.
- The application of nanoparticles in the secondary loop improves heat transfer within the evaporator of the district cooling unit and can lead to a reduction in the required heat transfer surface of the heat exchanger. This outcome can help designers and engineers improve the size and volume of the evaporator used in the chiller.

This study shows that nanofluids can have a significant impact when used in the HVAC systems, a reduction in pump and compressor work, enhanced cooling output and improved COP are all benefits of using these fluids. As earlier stated this can lead to improved system design as these systems can now become more compact thereby lead to reduced cost, and the energy savings obtained as a result of its use can lead to a reduction in the carbon emission associated with electricity production. Future works on this should involve a pilot-scale study as well as observing the wider effect of nanoparticles on a  district cooling network. We expect that this would reveal some other physical quantities and benefits not accounted for in this theoretical study.

## List of symbols

A: Area (m^2^)
C_p_: Specific heat capacity (kJ/kg·C)
D: Diameter of tube (m)
E: Exergy (W)
*f*: Friction factor
h: Heat transfer coefficient (W/m^2^C)
k: Thermal conductivity (W/mC)
L: Length (m)
$\dot{m}$: Mass flow rate (kg/s)
Nu: Nusselt number
P: Pressure (kPa)
Pr: Prandtl number
Q: Heat flux (W)
Re: Reynolds number
T: Temperature (C)
T_avg_: (T_5_ + T_6_)/2
U: Heat transfer coefficient (W/m^2^C)
*v*: Velocity (m/s)
*V*_*f*_: Volume flow rate (l/min)
W: Work (W)

## Greek symbols

∆P: Pressure drop (Pa)
η: Efficiency
μ: Dynamic viscosity (Pa s)
ρ: Density (kg/m^3^)
φ: Volumetric fraction of nanoparticles

## Subscript/superscript

0: Ambient
air: Air
bf: Base fluid
blower: Blower
c: Compressor
ci: Cold inlet
co: Cold outlet
cool: Cool
elect: Electrical
evp: Evaporator
ex: Exergy
f: Fluid
h: Hot fluid (shell side)
hi: Hot inlet
ho: Hot outlet
i: Inside (shell side)
i, coil: Inside cooling coil
LMTD: Log mean temperature difference
nf: Nanofluids
np: Nanoparticles
o: Outer (shell side)
p: Pump
s: Shell side
source: Source
t: Tube

## Abbreviations

AC: Air conditioning
AHU: Air handling unit
COP: Coefficient of performance
EER: Energy efficiency ratio
EES: Engineering equation solver
EG: Ethylene glycol
FCU: Fan coil unit
HVAC: Heating, ventilation, and air conditioning
RPM: Revolutions per minute
VCS: Vapour compression systems
